# Evaluation of the Formulation Parameter-Dependent Redispersibility of API Nanoparticles from Fluid Bed Granules

**DOI:** 10.3390/pharmaceutics14081688

**Published:** 2022-08-13

**Authors:** Martin Wewers, Jan Henrik Finke, Stefan Czyz, Bernard Van Eerdenbrugh, Edgar John, Guido Büch, Michael Juhnke, Heike Bunjes, Arno Kwade

**Affiliations:** 1 Institute for Particle Technology, Technische Universität Braunschweig, Volkmaroder Str. 5, 38104 Braunschweig, Germany; 2 Center of Pharmaceutical Engineering (PVZ), Technische Universität Braunschweig, 38106 Braunschweig, Germany; 3 Institute of Pharmaceutical Technology and Biopharmaceutics, Technische Universität Braunschweig, Mendelssohnstr. 1, 38106 Braunschweig, Germany; 4 Novartis Pharma AG, 4002 Basel, Switzerland

**Keywords:** wet media milling, nanosuspensions, fluidized bed granulation, redispersibility, formulation parameters

## Abstract

The production of nanosuspensions of poorly soluble active pharmaceutical ingredients (API) is a popular technique to counteract challenges regarding bioavailability of such active substances. A subsequent drying of the nanosuspensions is advantageous to improve the long-term stability and the further processing into solid oral dosage forms. However, associated drying operations are critical, especially with regard to nanoparticle growth, loss in redispersibility and associated compromised bioavailability. This work extends a previous study regarding the applicability of an API (itraconazole) nanosuspension as a granulation liquid in a fluidized bed process with focus on the influence of applied formulation parameters on the structure of obtained nanoparticle-loaded granules and their nanoparticle redispersibility. Generally, a higher dissolution rate of the carrier material (glass beads, lactose, mannitol or sucrose) and a higher content of a matrix former/hydrophilic polymer (PVP/VA or HPMC) in the granulation liquid resulted in the formation of coarser and more porous granules with improved nanoparticle redispersibility. HPMC was found to have advantages as a polymer compared with PVP/VA. In general, a better redispersibility of the nanoparticles from the granules could be associated with better dispersion of the API nanoparticles at the surface of the granules as deduced from the thickness of nanoparticle-loaded layers around the granules. The layer thickness on granules was assessed by means of confocal Raman microscopy. Finally, the dispersion of the nanoparticles in the granule layers was exemplarily described by calculation of theoretical mean nanoparticle distances in the granule layers and was correlated with data obtained from redispersibility studies.

## 1. Introduction

A significant proportion of novel drug candidates belongs to class II of the biopharmaceutical classification system (BCS) [1,2,3]. The corresponding active pharmaceutical ingredients (API) are characterized by a poor aqueous solubility and a high intestinal permeability. Consequently, the bioavailability of BSC II drugs is limited by their dissolution/solubility in the gastrointestinal tract, as they are absorbed rapidly once present in the dissolved state. By implication, the bioavailability of the corresponding APIs may be increased by increasing their dissolution rate [3]. One established method for this purpose is the reduction of the particle size of the APIs into the submicron size range, e.g., by wet media milling. The associated increase in the specific surface area elevates the dissolution rate, as described by Noyes-Whitney [4] and Nernst-Brunner [5,6], and thus increases the bioavailability.

Although API nanosuspensions are stabilized by one or more surface active substances (e.g., polymers and/or surfactants), they represent thermodynamically instable systems that tend to decrease their inherent free energy by decreasing the interfacial area with the dispersion medium, e.g., by agglomeration or ripening phenomena [7]. The resulting increase in particle/agglomerate size is diametrical to the initial objective of maximizing the surface area available for dissolution and can be assumed to lead to a deterioration of dissolution properties and finally bioavailability [7,8,9]. In order to overcome these challenges and to increase the therapeutic compliance of the patients, API nanosuspensions are often further processed to solid drug products by suitable drying methods.

Generally, spray drying [10,11,12,13,14] and freeze drying [12,13,14] are often described in this regard. However, as these processes lead to products often exhibiting powder characteristics unfavorable for further downstreaming into solid oral dosage forms (e.g., low bulk density and/or poor flowability [15,16,17]), drying of API nanosuspensions in fluidized bed processes is an appealing alternative. Corresponding products show enhanced flow, reproducible bulk density, decrease in dust, improved control of volumetric dosing operations and improved deaeration upon tablet compression [18,19]. API nanosuspensions containing hydrophilic polymers can be processed as spraying liquid in a fluid bed process. The hydrophilic polymer functions as the binder liquid in the fluid bed process. However, during the conversion of an API nanosuspension into the solid state by a respective drying operation, it is crucial to preserve the particle size of the originally applied nanosuspension after redispersion of the fluid bed granules in order to maintain an elevated dissolution rate of the poorly soluble API.

Generally, using higher mass ratios of matrix former/drying excipients (e.g., sugars or polymers) to the API is described to be advantageous in terms of nanoparticle redispersibility upon reconstitution of the drug products [10,11,14,20] and/or achieving high dissolution rates of the API [11,21,22]. These observations may be attributed to a higher degree of nanoparticle embedding or rather nanoparticle dispersity within the matrix material reducing potential interaction between individual nanoparticles and, finally, the probability of nanoparticle agglomeration [11,20]. This relationship between the nanoparticle size and dispersity within a solid oral dosage form was exemplarily described by Steiner et al. [23], estimating the theoretical mean nanoparticle distances within orodispersible films. The authors described that the redispersibility of nanoparticle-loaded orodispersible films improved with higher theoretical particle distances.

A former study of the authors [20] showed that the redispersibility of naproxen nanoparticles from nanoparticle-loaded granules obtained via a fluidized bed granulation process was highly dependent on the applied formulation parameters (e.g., carrier material, type and amount of additional polymeric drying excipient). Furthermore, the redispersibility could be qualitatively correlated with the embedding of the nanoparticles on the surface of the product granules. This led to the assumption that an increased embedding of nanoparticles results in enhanced redispersibility properties due to the dispersion of the nanoparticles in a larger volume of matrix material. The latter results in a decreased potential for nanoparticle interaction and consequently agglomeration during the drying process or in the dry product.

In consequence, the current study particularly focuses on the evaluation of formulation parameter-dependent differences in the dispersity of nanoparticles in granules and the transferability to another API. For this purpose, a nanosuspensions containing itraconazole was sprayed as the granulation liquid during a fluidized bed granulation process. The influence of the applied formulation parameters (i.e., carrier particle type, amount and type of matrix former/hydrophilic polymer in the nanosuspension) on the structure and the redispersibility of the resulting granules was of particular interest. Furthermore, the approach of Steiner et al. [23] for calculating theoretical particle distances between API nanoparticles was applied in order to exemplarily describe the relationship between the morphology of product granules or rather state of dispersity of incorporated nanoparticles and their redispersibility. In this regard, a novel technique for evaluating the structure of thin nanoparticle-loaded layers on granules by confocal Raman spectroscopy is introduced, which presents a promising tool for facilitating the understanding of the structure–property relationship of solidified nanoparticle-containing composites.

## 2. Materials and Methods

### 2.1. Materials

In the present study, micronized itraconazole (kindly provided by Novartis Pharma AG, Basel, Switzerland) was used as a poorly water-soluble model API. During milling, either a vinylpyrrolidone–vinyl acetate copolymer (PVP/VA, Kollidon VA 64, gift from BASF SE, Ludwigshafen am Rhein, Germany) or hydroxypropyl methyl cellulose (HPMC, Pharmacoat 603, gift from Shin-Etsu Chemical, Chiyoda, Japan) was used as a steric stabilizer in combination with sodium dodecyl sulfate (SDS, Sigma Aldrich, St. Lois, MO, USA). The same polymers PVP/VA and HPMC were also used as additional polymeric excipients during fluidized bed granulation.

**Figure 1:**
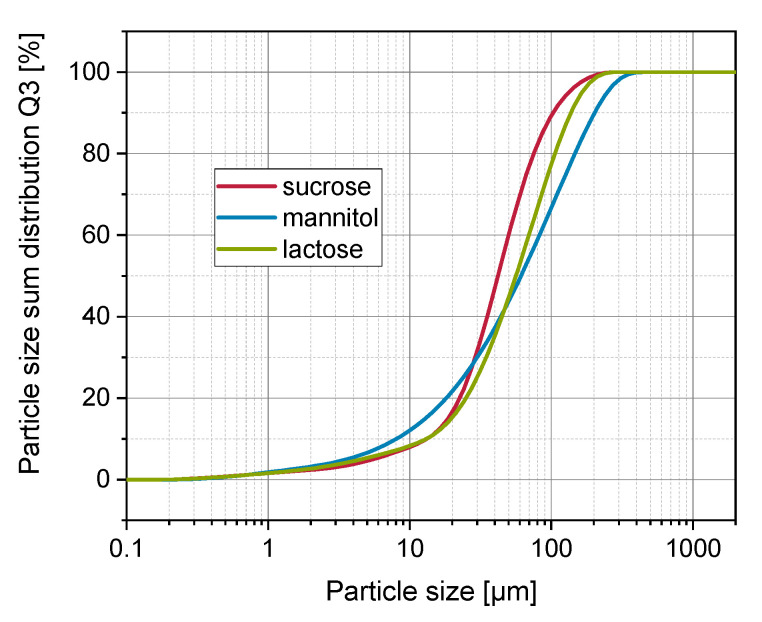
Particle size distribution of soluble carrier materials measured by means of laser diffraction (Mastersizer 3000, Malvern Panalytical, Malvern, UK) applying dry dispersion at 4 bar air pressure.

Sucrose (Nordzucker AG, Braunschweig, Germany), mannitol (Pearlitol 160 C, Roquette Frères, Lestrem, France) and lactose monohydrate (lactose, Granulac 140, Meggle AG, Wasserburg am Inn, Germany) were used as soluble carrier materials during granulation trials. As the applied sucrose showed a significantly different mean particle size in comparison to mannitol and lactose, fines of sucrose were separated by sieving in order to harmonize the particle size of all investigated carrier materials. Sieving was carried out in batches of 100 g sucrose using an air jet sieve (e200 LS, Hosokawa Alpine AG, Augsburg, Germany) equipped with a 20 µm sieve for 15 min at a pressure of 4000 Pa. In order to remove the fines sufficiently, the procedure was repeated twice. The separated fines smaller than 20 µm were discharged, and sieve retention was applied as carrier material during granulation trials. For particle size distribution of soluble carrier materials, see Figure 1. Additionally, glass beads (SiLibeads SOLID 40–70 µm, Sigmund Lindner GmbH, Warmensteinach, Germany) were applied as insoluble model carrier material during the granulation process.

### 2.2. Production of Itraconazole Nanosuspensions

Milling of itraconazole was conducted in a stirred media mill (MiniCer, kind loan of Netzsch Feinmahltechnik GmbH, Selb, Germany) operated in recirculation mode with a constant mass flow of about 35 kg·h^−1^. The process temperature was set to 25 ± 1 °C at the outlet of the milling chamber, and the operating parameters were kept constant for the production of differently formulated nanosuspensions. The stirrer tip speed was set to 9 m·s^−1^ and yttrium stabilized zirconia grinding media (Sigmund Lindner GmbH, Warmensteinach, Germany) with a mean diameter of 350 µm were used. The grinding media filling ratio of the grinding chamber was set to 0.8. Micronized itraconazole was processed in an aqueous suspension with a mass concentration of 10%. For stabilization of the API particles against agglomeration, either 2.50 wt% PVP/VA or HPMC together with 0.25 wt% SDS was applied. To achieve a sufficiently small particle size of approx. 150 nm, the milling duration was set to 6 h for all formulations.

### 2.3. Fluidized Bed Granulation

The milled nanosuspensions were directly sprayed as granulation liquid in a fluidized bed granulation process as also described in [20]. Briefly, the suspensions were sprayed onto fluidized carrier materials in a fluidized bed unit (MiniGlatt, Glatt GmbH, Binzen, Germany) in top-spray configuration with a 0.5 mm nozzle. For all granulation trials, the same experimental procedure was used. The nanosuspensions were applied with a mean feed rate of approx. 1.5 g·min^−1^ at an atomization air pressure of 0.7 bar. The volume flow of the fluidization air was manually increased during granulation from 9 to 12 m^3^·h^−1^ to maintain a sufficient fluidization during the granulation process, and the inlet air temperature was concomitantly decreased from 70 to 65 °C to maintain a constant outlet air temperature of around 35 °C.

Formulation parameters were varied between the granulation trials. To achieve the lowest polymer to API ratio of 0.25 in the granules, 50 g of nanosuspension was sprayed onto 50 g of carrier materials without addition of further excipients. In order to increase the polymer to API ratio in the granules, additional amounts of polymeric excipient (either PVP/VA or HPMC) were dissolved in the nanosuspension prior to the granulation. The mass of sprayed nanosuspension increased accordingly. In order to maintain the theoretical API content in the granules (i.e., 8.87 wt%), corresponding masses of additional polymer were subtracted from the carrier material. As a result, polymer to API mass ratios from 0.250 (original nanosuspension) to 0.875 (PVP/VA) or 0.750 (HPMC) were obtained in the product granules. For further information regarding the calculation of the corresponding mass of the additional polymer in the suspension, the corresponding masses of the sprayed granulation liquid, and the reduced mass of the carrier particles, please see the previous publication [20].

### 2.4. Characterization of Materials and Granules

#### 2.4.1. Particle Size Measurement

Particle size measurements of API nanosuspensions (original or redispersed) were performed via dynamic light scattering (DLS) with a Zetasizer nano ZSP (Malvern Panalytical Ltd., Malvern, UK). Samples were adequately diluted with saturated and filtered (pore size: 0.2 µm) API solution and equilibrated for 300 s to 25 °C before measurement. Afterward, each sample was measured in triplicate with a measurement duration of 180 s for each run. Mean values of the z-average (z-avg.; and polydispersity index (PdI), please see Supplementary Information S1) were used as characteristic property of the nanosuspensions. The refractive index of the API, was determined by applying Saveyn’s multiple solvent approach [24] at a wavelength of 589.3 nm and a temperature of 25 °C with a multiwavelength refractometer (Abbemat MW, Anton Paar GmbH, Graz, Austria). The same device was used for the determination of the RI of the saturated and filtered API solutions used as a dilution medium during measurements. The corresponding viscosities of these solutions were determined by means of capillary viscosimetry (Ubbelohde viscometer 52503, Schott-Geräte GmbH, Mainz, Germany) at 25 °C. For calculation of the dynamic viscosity, the density of the dilution media was determined with a pulsed excitation density meter (DMA 46, Anton Paar GmbH, Graz, Austria) at 25 °C.

Carrier materials were characterized by means of dry laser diffraction (Mastersizer 3000 with an Aero S dispersing unit, Malvern Panalytical Ltd., Malvern, UK) regarding their particle size and specific surface area. Measurements were conducted in triplicate using a dispersing pressure of 2.5 bar, and data evaluation was performed applying the Fraunhofer theory. Specific surface areas were assessed from the particle size distribution by considering the solid densities ρ_solid_ of the carrier materials (see Section 2.4.4).

The particle size of the nanoparticle-loaded granules was characterized by means of dynamic image analysis (QICPIC equipped with a GRADIS dispersion unit, Sympatec GmbH, Clausthal-Zellerfeld, Germany) as the mean diameter of a circle with an equivalent projection area.

#### 2.4.2. Redispersibility from Granules

For characterizing the redispersibility of the API nanoparticles from the product granules, samples were dispersed in saturated and filtered itraconazole solution on a magnetic stirrer for 15 min to facilitate complete dissolution of soluble excipients (i.e., carrier materials, polymeric matrix former). This method was chosen to prevent the dissolution of the API and to keep its particles size as constant as possible by hindering premature and artificial dissolution of the API particles, which would have led to falsely better redispersibility. The resulting dispersions were measured by means of DLS (see Section 2.4.1), and mean particle sizes (i.e., z-avg.) were recorded. The redispersibility from nanoparticle-loaded granules was characterized by calculation of the redispersibility index (RDI) [20]. In brief, the RDI normalizes the mean particle size of the API suspension, which was redispersed from the granules to the mean particle size of the original nanosuspension (including additional amounts of polymeric excipients, if applicable), directly prior to drying. Here, not the particle size as obtained after milling of the nanosuspension was used, because the additional polymers in the nanosuspension increase the viscosity, generally resulting in larger (apparent) particle sizes measured by DLS [23]. Insoluble glass beads were separated from redispersed API particles by sedimentation for 30 s of the redispersed suspension before sampling and measurement.

#### 2.4.3. Viscosity Measurement

The viscosities of differently stabilized nanosuspensions were determined by means of rotational viscosimetry (HAAKE Rheostress 6000, Thermo Fisher Scientific Inc., Karlsruhe, Germany, equipped with a double gap geometry). Samples were equilibrated for 10 min at 25 °C and measured with a shear rate of 0–500 s^−1^. Dynamic viscosities were calculated from the linear part of the corresponding rheograms.

#### 2.4.4. Gas Pycnometry

Solid densities ρ_solid_ of the applied API, polymers and carrier materials were determined by means of helium pycnometry (Ultrapyc 1200e, Quantachrome GmbH & Co., Odelzhausen, Germany).

#### 2.4.5. Determination of Bulk and Tapped Densities

Bulk densities ρ_bulk_ of raw carrier materials and granules were determined by gently introducing approx. 15 mL of powder into a graduated cylinder as the ratio of the mass of the powder to the volume occupied. Afterward, the cylinder was mechanically tapped (volumetric analyzer; Erich Tschacher Laboratoriumsbedarf Bielefeld, Bielefeld, Germany) 2500 times for determination of the tapped density ρ_tapped_.

#### 2.4.6. Porosity of Nanoparticle-Loaded Granules

The porosity of nanoparticle-loaded granules was characterized by means of nitrogen ad-/desorption (ASAP 2020, Micromeritics Instrument Corporation, Norcross, GA, USA) in duplicate. Samples of approx. 3 g were degassed for at least 6 h at room temperature. Afterward, ad-/desorption studies were carried out by cooling the samples in liquid nitrogen. Obtained desorption data were evaluated by the model of Barrett, Joyner and Halenda (BJH) [25] to extract the pore size distributions. As this method is used to characterize mesoporosity [26], the cumulative pore size volume up to pore sizes of 50 nm was extracted from the pore size distributions and used as a characteristic value for the mesoporosity of the nanoparticle-loaded granules.

#### 2.4.7. Confocal Raman Microscopy

Confocal Raman Microscopy (Alpha 300R, WITec GmbH, Ulm, Germany) was used to determine the distribution of the API in the surface layer of the nanoparticle-loaded granules. Generally, Raman microscopy was conducted with an excitation wavelength of 532 nm, operated at a laser power of approx. 5.5 mW and an integration time of 0.1 s. Two-dimensional depth scans of the samples were conducted with a size of 10 × 10 µm and a step size of 0.2 µm using 100-fold magnification. Obtained data were identically post-processed using cosmic ray removal, noise filtering and background subtraction. Afterward, datasets were compared with spectra of the pure API in order to identify and visualize the API distribution in a Raman chemical map. The thickness of the nanoparticle-loaded layer was calculated by the obtained area information as a mean thickness over the scan width (further information regarding the method development can be found in the Supplementary Information S2).

## 3. Results and Discussion

### 3.1. Redispersibility of Nanoparticles from Granules

**Figure 2:**
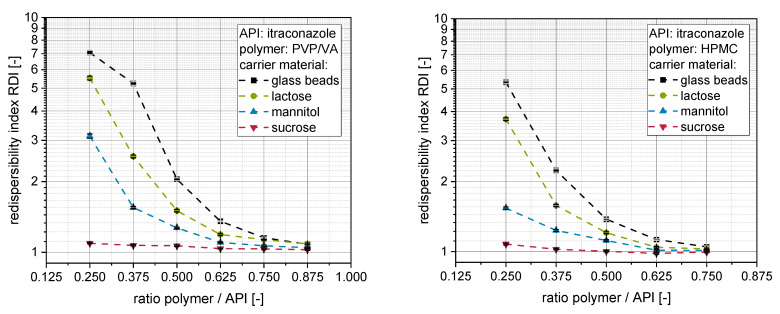
Redispersibility of nanoparticles from granules (*n* = 3) produced with glass beads, lactose, mannitol or sucrose as carrier materials as a function of the polymer to API (itraconazole) ratio in the sprayed nanosuspensions and in the product granules. PVP/VA (**left**) or HPMC (**right**) was used as a steric stabilizer in the nanosuspensions and as an additional hydrophilic polymer in the granules.

The outcome of the redispersibility studies for granules produced with itraconazole nanosuspensions stabilized either with PVP/VA (left) or HPMC (right) and different carrier materials (glass beads, lactose, mannitol, sucrose), as well as different amounts of additional polymeric excipient, can be seen in Figure 2.

Distinct dependencies of the redispersibility of the nanoparticles on the applied carrier material can be observed for both nanosuspensions. In accordance with previous investigations, sucrose performed best as a carrier material with the highest intrinsic dissolution rate (1.62 mg·cm^−2^·min^−1^), followed by mannitol (0.64 mg·cm^−2^·min^−1^) and lactose (0.29 mg·cm^−2^·min^−1^) [20]. In the present study, it can be additionally observed that granules produced with insoluble glass beads performed worst in the redispersibility studies compared to soluble carrier materials at a given polymer to API ratio. This further strengthens our hypothesis that the dissolution rate of the carrier material is a crucial material property during fluidized bed granulation of API nanosuspensions. It determines the degree of carrier particle dissolution and subsequent nanoparticle embedding upon resolidification of dissolved fractions during the drying process [20].

When applying an insoluble carrier material, only the polymeric excipients dissolved in the nanosuspension serve as embedding matrix material in the dry state. Consequently, larger amounts of additional polymeric excipient are necessary to achieve a redispersibility comparable to granules produced with soluble carrier materials. The necessary amount of additional excipient depends on the dissolution rate of the applied carrier material with a higher dissolution rate resulting in lower necessary amounts of additional excipient needed to completely maintain the nanoparticulate size and dispersity after redispersion.

However, besides the carrier material, the necessary amount of additional excipient in the nanosuspension to facilitate complete redispersibility from the granules is also dependent on the type of polymer applied. The redispersibility of granules produced with HPMC is in most cases better than the redispersibility of comparable (i.e., carrier material, ratio polymer to API) granules produced with PVP/VA, when the same carrier material and the same ratio polymer to API is processed. It is highly unlikely that the solid density of the applied polymers is significantly elevated over drying so that changes in dispersity of the nanoparticles could be explained by these. This can especially not explain the differences in redispersibility, as HPMC has a higher solid density (ρ_S,HPMC_ = 1.283 g·cm^−3^) than PVP/VA (ρ_S,PVP/VA_ = 1.153 g·cm^−3^), which accordingly results in a higher nanoparticle volume concentration for HPMC. Consequently, application of HPMC should result in an even less pronounced nanoparticle embedding in the surface layer of the granules. This should result in a higher potential of nanoparticle interaction and, consequently, agglomeration resulting in a compromised redispersibility [11,20,23].

The differences in redispersibility might possibly be attributed to different viscosities of the nanosuspensions prepared with the two different polymers as stabilizers and eventually as additional excipient (i.e., PVP/VA or HPMC). After milling, nanosuspensions stabilized with PVP/VA exhibit a far lower dynamic viscosity (ɳ_PVP/VA_ = 2.1 mPa·s) compared to the nanosuspension stabilized with HPMC (ɳ_HPMCV_ = 4.2 mPa·s). These differences might be even more pronounced when additional polymer is added to the nanosuspension as excipient before granulation. This in turn influences the granulation process and/or the formation of the nanoparticle-loaded layer around the carrier particles: Higher viscosities result in the formation of larger droplets upon spraying [27], which decreases the rate of evaporation of the granulation liquid’s solvent by the fluidization air, resulting in a stronger wetting of the carrier particles/granules by higher viscous granulation liquid [28]. As a result, material from the surface of the carrier particles may dissolve, depending on its dissolution rate, to a greater extent resulting in an enhanced nanoparticle embedding and redispersibility. Although this hypothesis could apply to the soluble carrier materials, it is not applicable for the insoluble glass beads used in this study and thus cannot explain the observed differences.

However, differences may be further explained by applying principles and mechanisms of particle engineering via spray drying and the concept of Peclet [29], which were already applied for explaining differences in redispersibility of spray dried nanosuspensions [30,31,32]. Larger droplets and/or a (viscosity-) associated decreased evaporation rate of the water within the granulation liquid may result in a slower recession of the droplet surface. This in turn may result in the different motion (e.g., diffusional) of the nanoparticles within the granulation liquid being fast compared to the receding droplet surface and in a rather homogeneous distribution of the nanoparticles in the solid state. For lower viscosities and higher evaporation rates, the receding droplet surface moves faster, resulting in an at least partial surface enrichment of the nanoparticles, which leads to nanoparticle agglomeration and consequently poor redispersibility [29]. Although this hypothesis is transferable to other scenarios influencing the effective drying surface of the applied granulation liquid (e.g., due to differences in wettability of the carrier materials by the differently stabilized nanosuspensions), it is challenging to validate it experimentally. However, a different drying behavior of the differently stabilized nanosuspensions may also lead to differences in the structure of the nanoparticle-loaded granules. Consequently, insights in the fundamental relationships of formulation parameter-associated differences in product performance may be extracted from the corresponding relationship of the structure and the quality property of the nanoparticle-loaded granules as discussed in the following.

### 3.2. Structure of Nanoparticle-Loaded Granules

**Figure 3:**
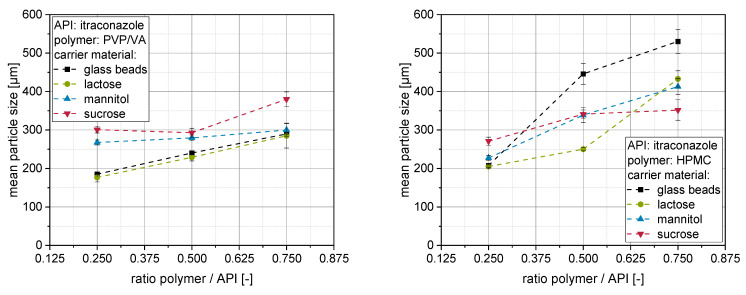
Mean particle size (*n* = 3) of nanoparticle-loaded granules containing PVP/VA (**left**) or HPMC (**right**) as stabilizing polymer and glass beads, lactose, mannitol or sucrose as carrier material.

The mean particle sizes of granules produced with differently stabilized itraconazole nanosuspensions and carrier materials can be found in Figure 3 (left: PVP/VA, right: HPMC) for polymer to API ratios of 0.25, 0.50 and 0.75.

Generally, it can be seen that the mean particle size of the granules increases with increasing polymer to API ratio in the nanosuspension. This observation is in accordance with the literature [22,28,33,34] and may be attributed to viscosity-related differences in droplet size [27,34], adhesiveness of the binder solutions [28] and/or wetting of the carrier surface by the granulation liquids [34]. As fluidized bed granulation is a combined coating/layering and granulation process, both mechanisms should be taken into account. The more prominent aspect leading to granule size growth is granulation, which is governed by the viscous properties and the binding properties of the granulation liquid.

For granules produced with nanosuspensions containing PVP/VA as a stabilizer and additional drying excipient, it can be observed that mean particle sizes of granules range between approx. 200 and 400 µm. Larger sizes of these granules generally coincide with higher redispersibilities (compare Figure 2), also valid between different applied carrier materials. Granules produced with insoluble glass beads or poorly soluble lactose exhibit the lowest mean particle sizes in comparison to mannitol and sucrose carrier particles exhibiting higher intrinsic dissolution rates [20]. This indicates that the partial surface dissolution and subsequent resolidification of the carrier materials does not only determine the degree of nanoparticle embedding on the product granules [20] but also may enhance granulation efficiency by promoting the formation and/or strengthening of solid bridges between individual carrier particles [35].

For granules stabilized with an HPMC-based nanosuspension with higher polymer concentrations, the mean particle sizes were higher when insoluble glass particles or poorly soluble lactose particles were applied as carrier materials (Figure 3 right). Here, the application of glass beads yielded the largest granules with particle sizes of up to about 500 µm. In contrast to that, granules prepared from mannitol or sucrose with HPMC were in the same size range as with PVP/VA or slightly smaller when the lowest polymer concentration was applied. In summary, the results indicate that HPMC may perform better as a binder by forming larger granules in a fluidized granulation process, which might be attributed to its formation of stronger solid bridges. It additionally indicates that the granulation aspect is more pronounced with a high ratio of HPMC as compared with the layering/coating aspect of fluidized bed granulation. However, the superiority of the granulation capacity of HPMC recedes into the background when the layering aspect, increasing with the solubility of the carrier material, is more pronounced. This results in comparable granule sizes for sugar carriers for both polymers.

**Figure 4:**
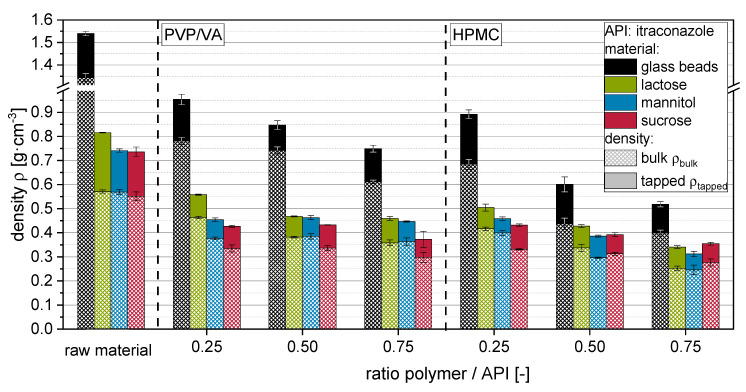
Bulk (ρ_bulk_) and tapped (ρ_tapped_) densities of raw carrier materials (i.e., glass beads, lactose, mannitol, sucrose) and corresponding nanoparticle-loaded granules produced with itraconazole nanosuspensions with different ratios of polymer (either PVP/VA or HPMC) to API.

Generally, the bulk density ρ_bulk_ of product granules correlates with data obtained from particle size measurements (Figure 4). Bulk densities decreased with increasing granule size (i.e., increasing polymer to API ratio) in comparison to the unprocessed raw materials. Here, an additional influence of the particle shape on the packing behavior and, by that, the interparticulate porosity cannot be excluded. Thus, ρ_tapped_ should be discussed more explicitly, as here the interparticulate porosity, caused, e.g., by differences in particle size or shape, is reduced to a minimum. Consequently, the corresponding values should at least partly reflect the differences in intraparticulate density/porosity of the granules and the additive contribution of interparticulate porosity originating from the particle size distribution and the deviation of particle morphology from a sphere. In this regard, the porosity increase in the corresponding particles Δε (raw materials or granules) was estimated by the difference in the porosity of the tapped powder bed ε_tapped_ and the porosity of a densest sphere packing ε_DSP,_ equal to 0.26 (Equation (1)).

(1) $$\Delta \varepsilon =\varepsilon_{\mathrm{tapped}}-\varepsilon_{\mathrm{DSP}}=\left(1-\frac{\rho_{\mathrm{tapped}}}{\rho_{s}}\right)-\varepsilon_{\mathrm{DSP}}$$

**Figure 5:**
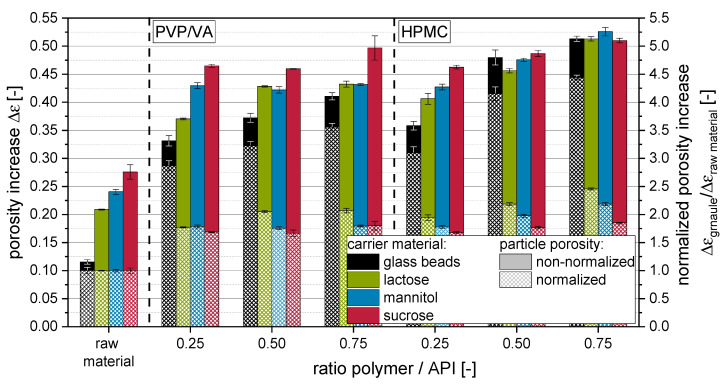
Porosity increase Δε of raw carrier materials (i.e., glass beads, lactose, mannitol, sucrose) and corresponding granules produced with an itraconazole nanosuspension stabilized with PVP/VA or HPMC and different ratios of polymer to API.

The solid densities ρ_S_ of the granules were calculated by considering the solid densities of the incorporated raw materials with the corresponding proportions. Values for the porosity increase Δε of the raw materials and granules are depicted in Figure 5. Additionally, the Δε of the granules is depicted after normalization to the Δε of the raw materials in order to accentuate the influence of the applied amount and type of polymer on the porosity of the granules.

Data for tapped densities and derived porosity increase Δε indicate that more porous and/or irregularly shaped granules are formed when more polymer is applied with the granulation liquid. This is to be expected, as the fluidized bed granulation is generally combining the layering/coating of primary carrier particle surfaces and the granulation, i.e., gluing a number of primary carrier particles together. The latter sub-process is adding to the formation of porous granule structures, as this developing interparticulate porosity is fixed within the granule. Additionally, the outer morphology of the granules is different to that of the starting material, as primary particles are glued together in a structure often compared with that of a raspberry, presenting a rough surface. Changes in the porosity increase are more explicit for HPMC than for PVP/VA. For the latter, especially granules produced with highly soluble carrier materials (i.e., mannitol and sucrose), show minor (ρ_tapped_) or no significant (Δε) dependencies on applied polymer to API ratios. Granules produced with nanosuspensions containing HPMC showed generally lower densities and higher porosities than comparable granules produced with PVP/VA, indicating that application of HPMC results in a more porous granule structure in comparison to granules produced with PVP/VA. However, it cannot be completely excluded for the soluble carrier materials that the dissolution and resolidification processes, which occur in dependence on the applied type and amount of polymer, can superimpose polymer-related differences in the granule porosity. However, data for insoluble glass beads show the same trends. The fact that differences are even more distinctive here may indicate that the dissolution and resolidification of soluble carrier materials during the granulation process may counteract a polymer amount- or type-associated increase in the porosity of granules.

**Figure 6:**
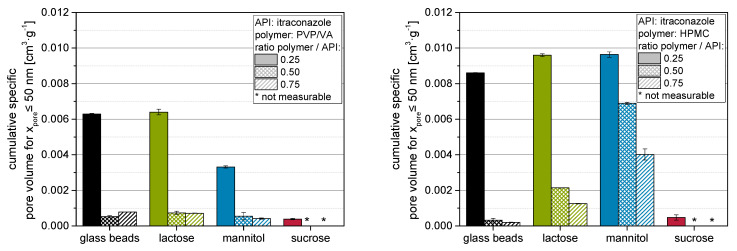
Results of BJH measurements of nanoparticle-loaded granules produced with itraconazole nanosuspensions containing different amounts of PVP/VA or HPMC as stabilizing polymers and glass beads, lactose, mannitol or sucrose as carrier material (*n* = 2, * refers to a detected porosity of 0).

As the porosity within the bulk of granules is constituted of different potential void spaces, it should be differentiated that (a) intragranular porosity may originate from (I) packing of primary particles within the granule as a result of the granulation process and (II) the porosity in the applied layer on the (primary) particle surfaces as a result of the layering/coating process. Conversely, the (b) intergranular porosity is mainly determined by the (III) granule size distribution and by the (IV) granule outer morphology, especially the surface roughness. The above introduced porosity increase takes all these factors cumulatively into account. The intragranular layer porosity (a-II) should possess the most direct effect on nanoparticle embedding, as it is most likely in the size range of the nanoparticles themselves, and it appears in the coating layer where the API nanoparticles are also situated. To this end, the porosity with pore sizes smaller than 50 nm in nanoparticle-loaded granules was evaluated with BJH measurements (Figure 6 left: PVP/VA; right: HPMC).

It is evident that in most cases, the mesoporosities (<50 nm, as determined by BJH measurements) of granules containing HPMC are considerably higher than those of granules containing PVP/VA for a comparable formulation (i.e., same carrier material and polymer to API ratio), but especially at the lowest polymer to API ratio of 0.25. Comparing these results to results presented in our previous study [20] and redispersibility of granules (see Section 2.1), it becomes clear that these differences may not originate from an insufficient nanoparticle embedding. Consequently, differences should be associated with a more pronounced pore structure within the nanoparticle-loaded layer and/or the associated layer formation mechanisms (e.g., drying behavior of the polymer). Contrarily to the porosity data shown above, the mesoporosity of the granules mostly decreases with increasing polymer to API ratio and by that higher polymer content in the granules. This can be attributed to the different size dimensions of the pores under consideration. The decrease in mesoporosity with increasing polymer to API ratio might be attributed to an increase in nanoparticle embedding at the carrier particle surface and/or a densification of the granules [36]. The BJH method does not access the complete intraparticulate porosity, especially that between the carrier particles, as it is limited to pore sizes in the lower nm range [25]. In contrast, the calculated porosities represent a more global intraparticulate porosity.

The observed differences in mesoporosity may be used to explain the influence of the polymer on the redispersibility of the nanoparticles from the granules. Higher (meso)porosities of granules produced with HPMC instead of PVP/VA might be related to an increased volume of the nanoparticle-loaded layer for granules produced with HPMC, which result in a better separation of the API nanoparticles within these layers and, consequently, in an enhanced redispersibility [11,20,23]. This hypothesis might also be assigned to a study from Figueroa and Bose [8], who similarly observed that granules with a higher porosity (determined via mercury intrusion) result in a better redispersibility of nanoparticles. However, when correlating redispersibility with porosity of corresponding granules, it has to be carefully distinguished between the different considered porosities to avoid misinterpretations.

### 3.3. Exemplary Description of Redispersibility of Nanoparticle-Loaded Granules

Results depicted in the previous sections and in our previous publication [20] indicate that redispersibility and structure of nanoparticle-loaded granules are highly dependent on the applied formulation parameters. Regarding redispersibility, it might be assumed that the volume, in which the API nanoparticles are dispersed in the dry state, is a critical factor influencing particle agglomeration and stabilization potential of individual nanoparticles after reconstitution [11,20,23]. In a previous study, it was already shown that the apparent thickness of nanoparticle-loaded layers around granules, assessed by means of confocal Raman microscopy, qualitatively differs for different formulations [20]. By further developing this method in the present study, the thickness of the nanoparticle-loaded layer h_layer_ was quantified by means of confocal Raman microscopy. Generally, it was observed that the thickness of the nanoparticle-loaded layer on the surface of the granules lies between approx. 1 and 3 µm (data not shown, see Supplementary Information S3). However, as not the thickness but rather the volume of the nanoparticle-loaded layers is more important regarding the degree of dispersity of the nanoparticles within these layers, microscopically derived nanoparticle-loaded layer volumes V_layer_ were calculated considering the specific surface area S_m,carrier_ (derived from particle size measurements, see Section 2.4.1) and the mass m_carrier_ of the applied carrier materials (Equation (2)).

(2) $$V_{\mathrm{layer}}=h_{\mathrm{layer}}\cdot S_{m,\mathrm{carrier}}\cdot m_{\mathrm{carrier}}$$

Theoretical layer volumes were calculated assuming no dissolution of the carrier material and ideal mixing of stabilizers. Accordingly, the actually applied API, polymer and SDS masses were divided by their respective solids densities to yield their volumes and were summed up to determine the theoretical layer volume.

**Figure 7:**
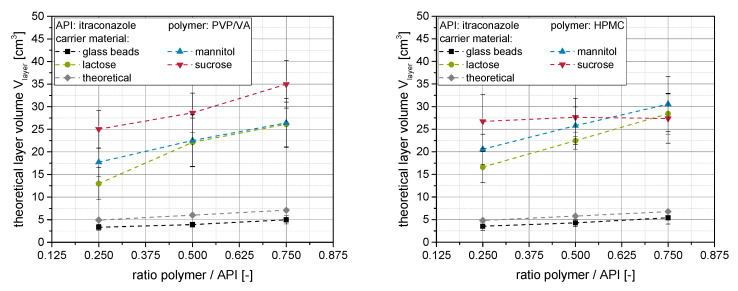
Microscopically derived nanoparticle layer volumes V_layer_ derived from specific surface areas of carrier starting materials and layer thickness determination by confocal Raman spectroscopy (*n* = 10) for granules produced with different carrier materials (glass beads, lactose, mannitol or sucrose) and an itraconazole nanosuspension stabilized with different amounts of PVP/VA (**left**) or HPMC (**right**).

The (actual) layer volumes, calculated using the measured layer thickness and Equation (2), can be seen in Figure 7 (left: PVP/VA, right: HPMC) as a function of the polymer to API ratio in the product granules.

Generally, it can be seen that the layer volumes increase to a higher or lesser extent with increasing polymer concentration within the granulation liquid, irrespective of the applied carrier material or polymer type. This was expected, as more material is present within the nanosuspension, which participates in the layer formation process and in which the nanoparticles can be dispersed upon resolidification. It can additionally be seen for both polymer types that the layer volume at a given polymer concentration depends on the applied carrier material. For soluble carrier materials, layer volumes are a multitude higher than the theoretical volumes (assuming no carrier dissolution) and align with the dissolution rates of the carrier materials [20]. The differences diminish at higher polymer to API ratios, indicating a superposition of layer formation processes by dissolved polymer, as well as dissolution and resolidification of the carrier material.

Layer volumes were the lowest on insoluble glass beads for both polymers, as with this carrier, the nanoparticle-layers are only formed by the granulation liquid and no surface dissolution takes place upon granulation. Here, the layer volumes were even slightly lower than theoretical volumes calculated from the applied spraying mass, indicating a slight loss of polymer within the granulation chamber. In principle, however, the layer volumes derived using the two different ways of calculation are in good agreement for this type of carrier underlining the validity of the approach. The application of HPMC tended to result in slightly higher layer volumes at a given polymer content in the granules (except for sucrose), which might be attributed to a higher porosity of the corresponding nanoparticle-loaded layers (see Section 3.2). However, differences diminish at higher polymer concentrations, further indicating a superposition of the different layer formation processes.

The data obtained for the layer volumes may be further used for exemplarily describing theoretical mean distances between individual nanoparticles within the nanoparticle-loaded layer around the granules. Assuming that the carrier particles and API nanoparticles are spherical with a uniform particle size, that the nanoparticle-loaded layer has no porosity and is homogeneously distributed on the surface of the carrier material, and that the API nanoparticles are uniformly distributed in this layer, the theoretical distances of the surfaces of nanoparticles within the layer a_theor._ can be calculated following the study of Steiner et al. [23] applying Equation (3).

(3) $$a_{\text{theor.}}=\sqrt[{3}]{x_{50,\mathrm{API}}^{3}\cdot \left(\frac{\left(1-\varepsilon_{\mathrm{DSP}}\right)\cdot V_{\mathrm{layer}}}{V_{\mathrm{API}}}\right)}-x_{50,\mathrm{API}}$$

**Figure 8:**
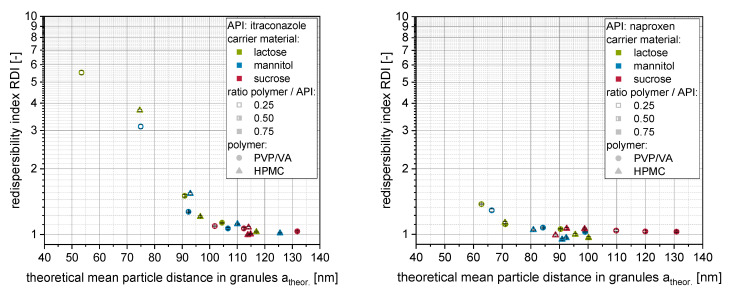
Correlation of the redispersibility index (RDI) with the theoretical mean nanoparticle distance for granules produced with itraconazole (**left**) or naproxen (**right**; data from [20]) nanosuspensions stabilized with different amounts of PVP/VA or HPMC and three different soluble carrier particles (lactose, mannitol or sucrose).

Here, x_50,API_ refers to the mean particle size (i.e., z-avg._orig_) of the API nanoparticles within the corresponding nanosuspension before granulation (including additional polymeric drying excipient, see Section 2.4.2.), ε_DSP_ to the porosity of a densest sphere packing, and V_API_ to the total volume of the API within the granules. The dependency of the redispersibility of nanoparticles from the granules (i.e., RDI) on the theoretical mean distances of the nanoparticles within the surface layer of the granules is presented in Figure 8 (left: itraconazole) for the soluble carrier materials. Additionally, granules with naproxen from our previous publication [20] were re-evaluated (Figure 8 right).

For soluble carrier materials (i.e., lactose, mannitol, sucrose), for each API, one consistent dependency of the redispersibility on the calculated mean nanoparticle distances can be observed irrespective of the exact formulation (i.e., type of polymer, ratio polymer to API). The granules were completely redispersible when theoretical mean distances of nanoparticles within the granules were larger than approx. 100 nm (itraconazole) and 80 nm (naproxen), respectively. These differences indicate that API-specific characteristics (e.g., particle shape, particle size distribution, physicochemical properties) influence the minimum mean nanoparticle distance that is sufficient for an appropriate redispersion. However, the minimum nanoparticle distances are in the same order of magnitude as those shown in the study of Steiner et al. [23] (particle distance of 150 nm for complete redispersibility considering the factor of ½ as particle center distances were calculated), highlighting the applicability of the corresponding approach to other nanoparticle-containing composites.

**Figure 9:**
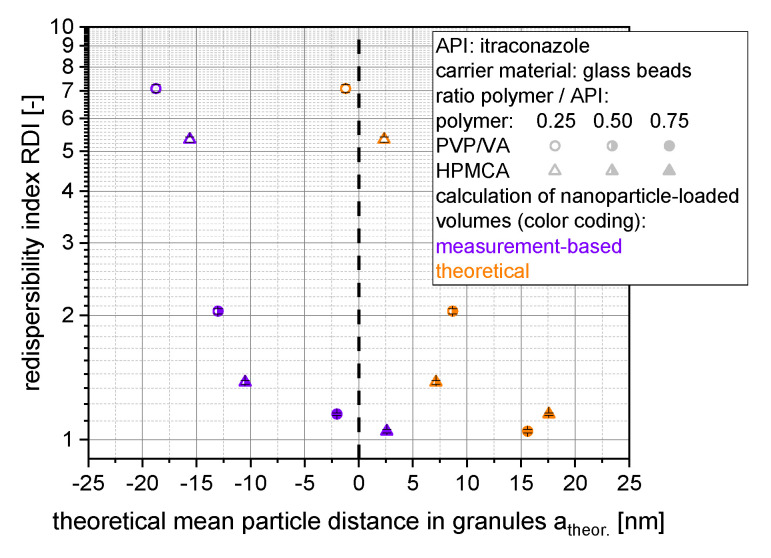
Correlation of the redispersibility index (RDI) with the mean theoretical nanoparticle distance for granules made out of insoluble glass beads as carrier material and an itraconazole nanosuspension stabilized with different amounts of PVP/VA or HPMC. Theoretical mean nanoparticle distances were calculated with theoretical and measurement-based nanoparticle-containing layer volumes.

The correlation of the redispersibility of nanoparticles from granules and the theoretical mean distances of the nanoparticles within the granules based on insoluble glass beads as carrier material can be seen in Figure 9.

Here, even negative distances were obtained when measured layer thickness/volumes were considered, which indicates deficient assumptions regarding the structure of the nanoparticle morphology, the nanoparticle-loaded granules and/or related choice of selected material parameters (e.g., specific surface area). Applying theoretically calculated layer volumes, particle distances were shifted toward positive particle distances in the lower nm range. However, similar dependency of the redispersibility on the theoretically calculated particle distances (irrespective of the applied layer volumes) highlights the importance of the degree of dispersity of the nanoparticles within the granules for their redispersibility.

## 4. Conclusions

The findings of the present study highlight the effects of formulation changes on the granules obtained from fluidized bed granulation using an itraconazole nanosuspension as granulation liquid. Variation of formulation parameters regarding the type of hydrophilic polymer (PVP/VA and HPMC), the ratio of polymer to API and the starting carrier material resulted in significant differences in redispersibility of nanoparticle-loaded granules. In this regard, the findings of a previous study [20] concerning the influence of the carrier material on naproxen-containing granules, as well as the applied type and amount of additional polymer in a naproxen nanosuspension, could be confirmed for an itraconazole nanosuspension. Additionally, the observations were extended by applying insoluble glass beads as a model carrier material. Corresponding data align with data obtained for soluble carrier materials (i.e., lactose, mannitol and sucrose), illustrating the importance of the carrier particle surface dissolution and subsequent resolidification. For soluble carrier materials, a high intrinsic dissolution rate of the carrier material facilitates the preservation of nanoparticle size and dispersity within the nanoparticle-loaded granules. Correspondingly, formulation development (e.g., with respect to the necessary amount of additional polymeric drying excipient) is supported by these findings. With respect to the latter, insoluble glass beads may represent the worst case concerning the redispersibility from granules at a given formulation, the necessary amount of additional polymeric drying excipient for maintaining a complete redispersibility and, consequently, regarding the theoretically achievable maximum drug loads in granules. The same differences for HPMC and PVP-containing formulations were observed for soluble and insoluble carrier materials. Accordingly, data for insoluble glass carrier particles further emphasize that enhanced redispersibility may not only be attributed to a more pronounced carrier particle dissolution and subsequent resolidification.

Thorough investigations of the structure of the obtained product granules generally showed that the application of carrier materials with a higher solubility and dissolution rate (glass beads < lactose < mannitol < sucrose), as well as higher contents of additional polymeric drying excipient in the granules resulted in the formation of coarser granules with a more porous structure in the macroporous (i.e., within the granules) as well as on the mesoporous range (i.e., within the API-containing surface layer). Redispersibility of the nanoparticles from the granules is generally improved with increasing theoretical nanoparticle distances. It can be facilitated by (a) higher intrinsic dissolution rate of the carrier, (b) higher concentration of stabilizing polymer, and (c) by the nature of the polymer. Regarding the latter, HPMC increases the volume of the API-containing surface layer in most cases by a more pronounced formation of pores. The presented method for estimating the mean nanoparticle distances can be applied for formulation prototyping and optimization during fluidized bed granulation process development for API nanosuspensions. However, it appears limited in its applicability to insoluble carriers such as glass. Future research should also address the possibilities to control carrier dissolution and pore formation by tuning the process parameters and, by that, process states (such as in-process humidity) in such processes.

## Data Availability

The data presented in this study are available on request from the corresponding author.

## Supplementary Materials

The following supporting information can be downloaded at: https://www.mdpi.com/article/10.3390/pharmaceutics14081688/s1.
