# Supplementary material for: Evaluation of the Formulation Parameter-Dependent Redispersibility of API Nanoparticles from Fluid Bed Granules
Source: Pharmaceutics. 2022 Aug 13;14(8):1688. doi: 10.3390/pharmaceutics14081688 (PMC9414476; doi:10.3390/pharmaceutics14081688)
Supplement: Supplementary file 1 [file pharmaceutics-14-01688-s001.zip › pharmaceutics-1846530-supplementary.pdf]

Supplementary Information

S1: Polydispersity indices (PdI) of redispersed nanosuspensions

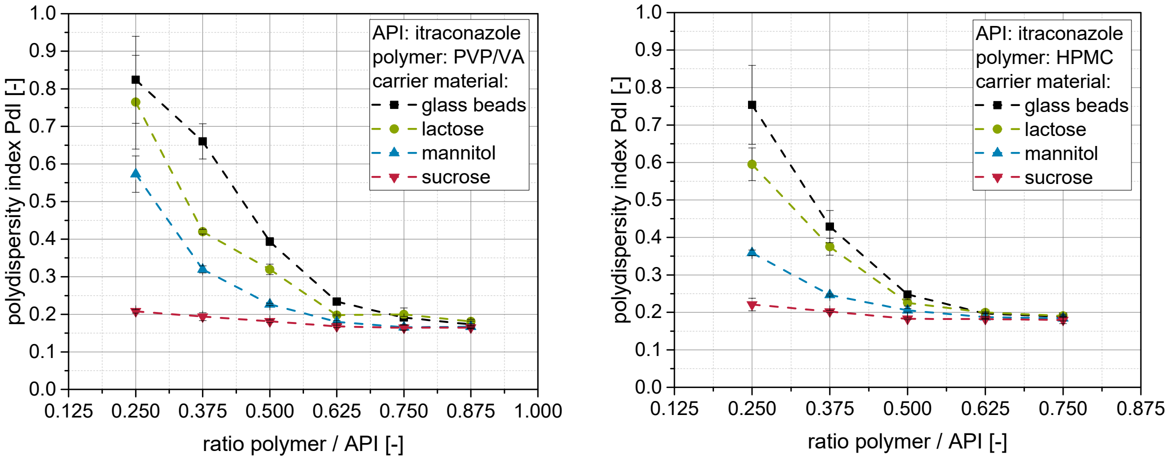

Figure S1. Polydispersity index (PdI) of suspensions redispersed from nanoparticle-loaded granules (n = 3) produced with glass beads, lactose, mannitol or sucrose as carrier materials as a function of the polymer to API (i.e. itraconazole) ratio in the applied nanosuspensions or in the product granules, respectively. PVP/VA (left) or HPMC (right) were used as steric stabilizer in the nanosuspensions and as additional drying excipient.

S2: Method development and validation of method for determination of the thickness of nanoparticle-loaded layers on granules

In order to obtain nanoparticle-containing coatings whose thickness can be easily assessed by conventional methods, laboratory glass substrates were coated with itraconazole nanosuspensions by a dip coating process. In order to achieve a variety of coating thicknesses, itraconazole nanosuspensions stabilized either with PVP/VA or HPMC (ratio polymer to API 0.25 and 0.50) and draw velocities of 100 and 300 mm·min^{-1} were applied during dip coating experiments using a laboratory dip-coater (Coater 4, IDLab, Czech Republic). After drying of the films for approx. 12 h at ambient conditions, a scratch was performed on the coating by dragging a stylus with a load of 500 g and a velocity of 8 mm·s^{-1} across the surface of the coating vertically to the coating direction.

The thickness of nanoparticle-loaded coatings was determined by a profilometric approach (DektakXT, Bruker Corporation, USA). A diamond stylus with a tip radius of 5 µm was drawn orthogonally over the scratch in the coating at mean height. The thickness of the coatings was afterwards calculated as the difference of the mean maximum and minimum height of the coating.

Coating thicknesses were additionally determined by means of confocal Raman microscopy as described in section 2.4.7. at mean height of the coatings. After postprocessing of data (i.e. cosmic ray removal, noise filtering and background subtraction) and comparison of datasets with spectra of pure itraconazole, threshold for CCD counts attributed to the API and consequently considered in layer thickness determination needed to be adapted. Here, values of 310 (PVP/VA) and 85 (HPMC) were chosen, as these values resulted in comparable results in determined layer thicknesses by both methods applied (see figure S2).

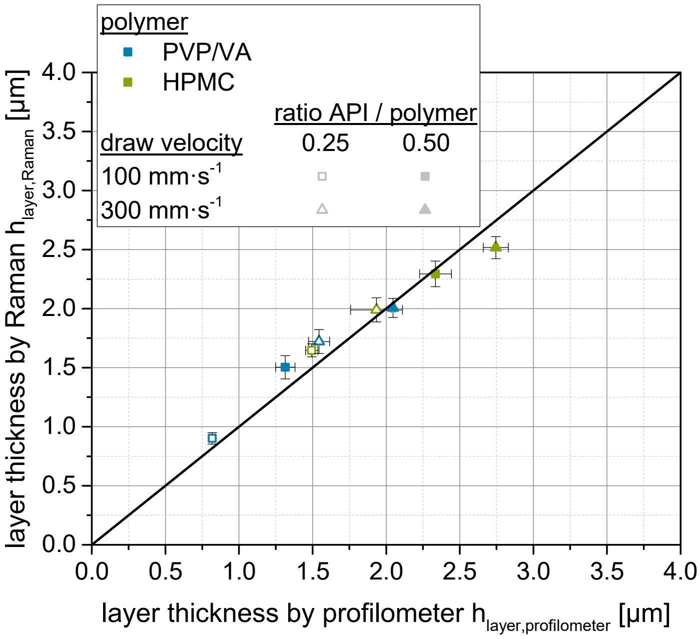

Figure S2. Parity plot of determined layer thickness by means of profilometry and confocal Raman microscopy for nanoparticle-loaded coatings produced by dip coating of an itraconazole nanosuspension stabilized with different amounts of PVP/VA or HPMC with different draw velocities on glass substrates.

The same procedure was applied for thickness determination of nanoparticle-loaded layers on granules. Although it cannot be evaluated whether the applied method resulted in definite representation of real layer thickness on granules, obtained trends are seen as reliable, as data were processed in a comparable and thorough manner.

S3: Thickness of nanoparticle-loaded layers on granules

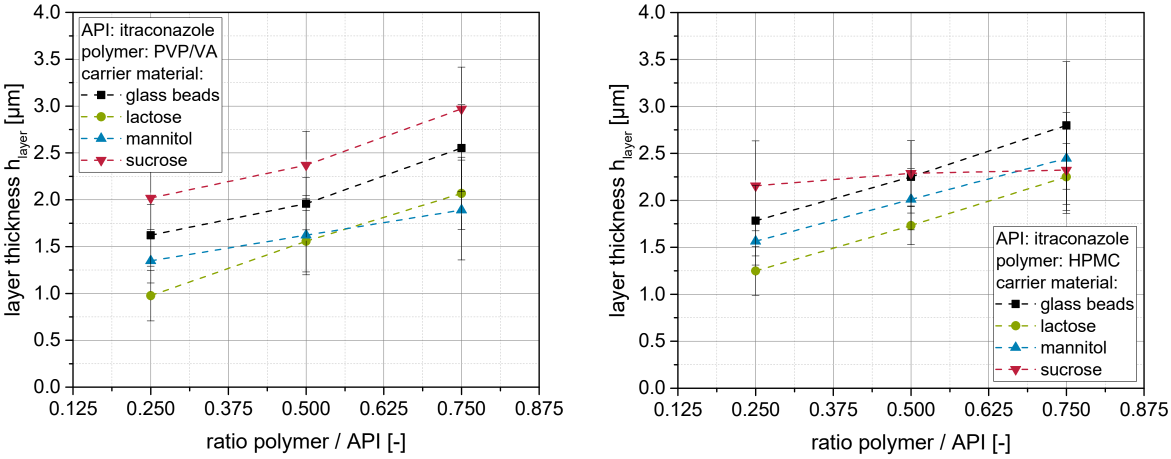

Figure S3. Thickness of nanoparticle-loaded layers on granules as determined by confocal Raman microscopy (n = 10) for granules produced with different carrier materials (glass beads, lactose, mannitol or sucrose) and itraconazole nanosuspensions stabilized with different amounts of PVP/VA (left) or HPMC (right).

Relatively high standard deviations in obtained layer thicknesses may be attributed to a broad assumed distribution of layer thicknesses on granules in combination with a small sample size (n = 10 granules) in comparison to the basic population ($m_{granules} \approx 50$ g).
